# Presentation of cytokine profile in relation to oxidative stress parameters in patients with severe COVID-19: a case-control pilot study

**DOI:** 10.12688/f1000research.55166.2

**Published:** 2021-10-14

**Authors:** Marija Petrushevska, Dragica Zendelovska, Emilija Atanasovska, Aleksandar Eftimov, Katerina Spasovska

**Affiliations:** 1 Institute of preclinical and clinical pharmacology and toxicology, University of Ss Cyril and Methodius, Faculty of Medicine, Skopje, Macedonia; 2 Institute of pathology, University of Ss Cyril and Methodius, Faculty of Medicine, Skopje, Macedonia; 3 University Clinic for Infectious Diseases and Febrile Conditions, Faculty of Medicine, University of Ss Cyril and Methodius, Skopje, Macedonia

**Keywords:** oxidative stress, COVID-19, cytokines

## Abstract

**Introduction: **COVID-19 can be worsened by hyper-production of cytokines accompanied by increased level of oxidative stress. The aim of this study was to investigate the correlation between a set of cytokines and the markers of the oxidative stress.

**Methods: **The levels of cytokines IL-2, IL-4, IL-6, IL8, IL-10, VEGF, IFN-γ, TNF-α, IL-1α, MCP-1 and EGF were determined by using High Sensitivity Evidence Investigator™ Biochip Array technology. The oxidative stress parameters (d-ROM, PAT, OS index) were measured in serum on FRAS5 analytical photometric system.

**Results: **IL-6, IL-8, IL-10, VEGF, MCP-1 and EGF were significantly higher (p<0.05) in the patients with severe COVID-19 with increased levels of IL-2, IFN-y, TNF-α and IL-1α. The d-ROM, OS index, and PAT were significantly higher (p<0.05) in severe COVID-19 patients. IL-6 demonstrated the strongest correlation with all of the markers of the oxidative stress, d-ROM (r=0.9725, p=0.0001), PAT (r=0.5000, p=0.0001) and OS index (r=0.9593, p=0.012). Similar behavior was evidenced between IFN-y and d-ROM (r=0.4006, p=0.0001), PAT (r=0.6030, p=0.0001) and OS index (r=0.4298, p=0.012).

**Conclusion: **The oxidative stress markers show good correlation with the tested cytokines which can be measured at the beginning of the disease in a primary care setting to predict the course of COVID-19.

## 1. Introduction

Cytokine storm syndrome has been widely discussed and proposed as one of the underlying aetiologies of respiratory failure in patients infected with SARS-CoV-2. Pro-inflammatory cytokines play a key role in large number of respiratory viral infections by activation of the adaptive immune response and, when this response is not controlled, it can lead to involvement of the lung tissue in the course of ARDS or can result in severe damages of multiple organs. For example, following influenza viral infection, an excessive amount of reactive oxygen species (ROS) is produced in several tissues including alveolar epithelium and endothelium ^1^ for which induced expression of cytokines through activation of Toll-like receptors (TLR3, TLR7 and TLR8, retinoic acid inducible gene I and members of NOD-like receptor family) stand in the background of the pathogenesis. ^2, 3^ Oxidative stress is typical for infection of human respiratory syncytial virus, ^4^ rhinoviruses, ^5^ and many other viruses. This has been discussed in previously published reviews ^7- 12^ and as well, several experimental studies suggest that cytokine storm correlated with direct tissue injury and lead to unfavourable prognosis of severe form of the COVID-19 disease. ^7^ Briefly, particularly high levels of IL-6, IL-10, IL-2R and TNF-α have been reported in patients with severe form of the disease ^13, 14^ although other authors suggest that more cytokines, such IL-1β, IL-1RA, IL-8, IL-18 are included in the COVID-19 pathogenesis. ^7, 13, 14^

Authors have suggested that the innate immune response follows same pathway for SARS-CoV-2 infection. ^1, 8^ Namely, ROS is a strong ligand and a direct mediator in the NLPR3 (inflammasome) trigger. Moreover, NF-κB, which is activated by ROS, triggers transcriptional levels of NLPR3 are enhanced by TLR and NLR ligands. This means that the inflammasome is increased by ROS either directly or indirectly. ^8, 12^ To the addition of ROS, H _2_O _2_ activates NF-κB to produce inflammatory cytokines. ^15^ Hyperproduction of IL-6, TNF-α, IL-1β, IP-10, GCSF, MCP-1, MIP1-α/CCL3 and elevated blood ferritin are also observed in patients infected with SARS-CoV-2. ^7, 16^

For this purpose, and in the light to share more experimental data as evidence to the suggested pathogenesis of COVID-19 with the scientific community, we have utilized a highly standardized cytokine assay to measure plasma levels of 11 inflammatory cytokines potentially associated as key factors with the cytokine storm syndrome. Afterwards, we have investigated which of these cytokines involved in the cytokine storm of COVID-19 show good association/correlation with the oxidative stress markers determined with fast and inexpensive photometric analytical method. Moreover, the relation between the cytokines, oxidative stress markers and the most commonly used inflammation-related biomarkers (CRP, D-dimers, PLR, NLR and LDH) in severe form of the disease was investigated.

## 2. Methods

### 2.1 Study design, patients profile and data collection

**Figure 1.**
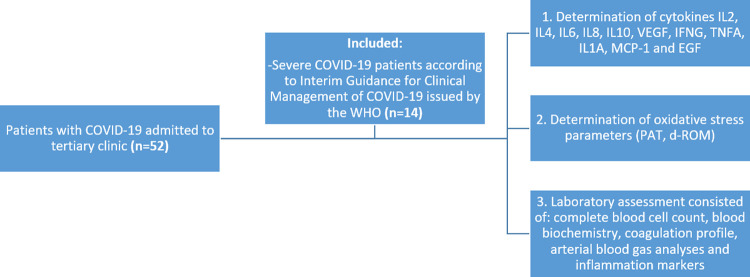
Flow-chart of the study.

52 patients with COVID-19 were hospitalized at the University Clinic for Infectious Diseases and Febrile Conditions, Skopje, Republic of North Macedonia at the beginning of the pandemic within a period of 1 month. 14 patients classified with severe COVID-19 (nine males and five females) with a mean age of 58.36 years (range from 36 to 71 years) were included in this study (study group). The diagnosis and classification of COVID-19 were based on the Interim Guidance for Clinical Management of COVID-19 issued by WHO. Severe cases in addition to severe pneumonia met at least one of the following conditions: SpO _2_ <90% on room air, respiratory rate >30 breaths/minute or presence of severe respiratory distress. All patients were confirmed to have SARS-CoV-2 infection by real-time reverse transcriptase-polymerase chain reaction assay (RT-PCR). Severe form of COVID-19 as primary exposure variable, demographic characteristics, medical history, clinical symptoms and signs, concomitant medication, outcome data, as well as laboratory analyzes were obtained from the patients’ medical records were other predictor variables. For purpose of control and comparison between groups, we have analyzed samples of 20 healthy individuals with negative RT-PCR test for SARS-CoV-2 (12 males and eight females, mean age 54).  This group had no defined risk factors, were in good health as determined by the medical history, complete physical examination, electrocardiogram (ECG), and clinical laboratory tests. The female individuals had negative serum pregnancy test and absence of lactation. The study flow chart is shown in Figure 1. The study was approved by the local ethics committee (Ethics Committee of the Faculty of Medicine, University of Ss Cyril and Methodius, Skopje, Republic of North Macedonia, No #03-366/7) and complies with the STrengthening the Reporting of Observational studies in Epidemiology (STROBE) statements for reporting of observational trials. ^17^

### 2.2 Method for determination of d-ROMs, PAT and oxidative stress index

PAT (total antioxidant power, iron reducing) and d-ROMs (plasma peroxides) were measured on a FRAS5 analytical photometric system (H&D, Italy). Samples were collected and analyzed immediately after hospital admission. The instructions of the manufacturer were followed for the both tests. The d-ROM and PAT are reported in equivalents of H _2_O _2_ and ascorbic acid, respectively. Oxidative stress index (OSI) presents information obtained from d-ROMs Fast test and the PAT test that is automatically calculated by the manufacturer’s software (OB manager, FRAS5, H&D, Italy) with normal reference values less than 40.

### 2.3 Cytokines profile assay

The High Sensitivity Evidence Investigator™ Biochip Array technology (Randox Laboratories, GB) was used to perform simultaneous quantitative detection of multiple analytes from a single patient sample (14 SARS-CoV-2 infected and 20 non-infected individuals).

100 μL of plasma was used in biochip carriers, following by incubation on thermo-shaker for 1 hour at 37°C and 370 rpm and 16–20 hours incubation at 4°C. Afterwards, carry out of two wash cycles and 300 μL conjugate was added into each well followed by another incubation of 1 hour at 37°C and 370 rpm. At the final step after twice washing the carriers, fluorescent dye was added to carriers according to protocol and carriers were captured by Evidence Investigator Array. Results were processed automatically using EvInvest software and levels of cytokines IL-2, IL-4, IL-6, IL-8, IL-10, VEGF, IFN-γ, TNF-α, IL-1α, MCP-1 and EGF were calculated as pg/mL.

### 2.4 Statistical analysis

Exposure variables were summarized using descriptive statistics. Data were described as number and/or percentage, or median and range or mean and standard deviation (SD) or standard error of mean (SEM), where appropriate. Differences between groups were explored using the t-test followed by Mann–Whitney where appropriate. A p-value less than 0.05 was considered significant. Spearman r coefficient of correlation was performed. All analyses were made using the statistical program GraphPad Prism 9 (USA) (RRID:SCR_000306); an open-access alternative is JASP (RRID:SCR_015823).

## 3. Results

### 3.1 Demographics and laboratory findings

All 14 patients with a mean age of 58.36 years had severe form of the disease. The average time from onset of symptoms to hospital admission was 10.52±2.33 days (range 7–16 days). All of them had underlying medical conditions at admission. The most frequently reported comorbidities were hypertension, diabetes and chronic cardiac disease. The most prominent and disturbing symptoms reported by the patients on admission were high body temperature (80%), dyspnea (64%), malaise (62%) and cough (56%). The mean value of all clinical laboratory parameters upon hospitalization are presented in Table 1. Abnormal values for CRP, LDH, PLR, D-dimer and NLR were observed. The mean ± SEM value for CRP was 144.7 ± 21.37 mg/L, LDH was 823.4 ± 80.02 IU/L, PLR was 538.2 ± 85.09, NLR was 17.08 ± 2.058, and D-dimer was 2688 ± 499.1 ng/mL. All 14 patients had increased values for ALT, AST and WBC in comparison to the individuals not infected with SARS-CoV-2. The observed statistically difference between the two groups was significant in all cases (p < 0.05).

**Table 1.** Laboratory findings in severe COVID-19 patients and non-infected individuals expressed as mean ± SEM.

Parameter	Severe COVID-19 patients mean ± SEM (n = 14)	Not infected individuals mean ± SEM (n = 20)	p (t-test)
IL-6 (pg/mL)	250.1 ± 39.07	2.135 ± 0.453	**0.0001**
IL-2 (pg/mL)	4.426 ± 2.177	2.005 ± 0.402	0.2818
IL-4 (pg/mL)	1.936 ± 0.268	1.956 ± 0.137	0.3150
IL-8 (pg/mL)	108 ± 19.79	7.159 ± 1.298	**0.0001**
IL-10 (pg/mL)	11.14 ± 4.551	0.916 ± 0.219	**0.0001**
VEGF (pg/mL)	530.7 ± 147.1	27.04 ± 4.708	**0.0001**
IFN-g (pg/mL)	1.487 ± 0.745	0.389 ± 0.082	0.3889
TNF-a (pg/mL)	5.223 ± 0.751	3.646 ± 0.757	0.090
IL-1a (pg/mL)	0.4614 ± 0.263	0.2153 ± 0.0422	0.7210
MCP-1 (pg/mL)	891 ± 92.35	89.61 ± 12.18	**0.0001**
EGF (pg/mL)	65.37 ± 17.46	24.28 ± 5.367	**0.0318**
d-ROM (U.Carr)	448.8 ± 30.37	271 ± 5.590	**0.0001**
PAT (U.Carr)	3048 ± 100.1	2406 ± 71.55	**0.0001**
OSI	107.7 ± 14.38	21 ± 2.527	**0.0001**
CRP (mg/L)	144.7 ± 21.38	2.1 ± 0.05	**0.0001**
LDH (IU/L)	823.4 ± 80.02	156 ± 20.31	**0.0001**
NLR	17.08 ± 2.058	1.5 ± 0.02	**0.0001**
PLR	538.2 ± 85.09	113 ± 10.35	**0.0001**
D-dimer (ng/mL)	2688 ± 499.1	225 ± 22.75	**0.0001**
WBC (×10 ^3^μL)	14 ± 2.004	6.1 ± 1.365	**0.0019**
ALT (U/L)	51.93 ± 7.171	28.96 ± 2.658	**0.0018**
AST (U/L)	61.210 ± 7.283	30.56 ± 3.487	**0.0002**

### 3.2 Cytokine profile, oxidative stress parameters and commonly used biomarkers

**Figure 2.**
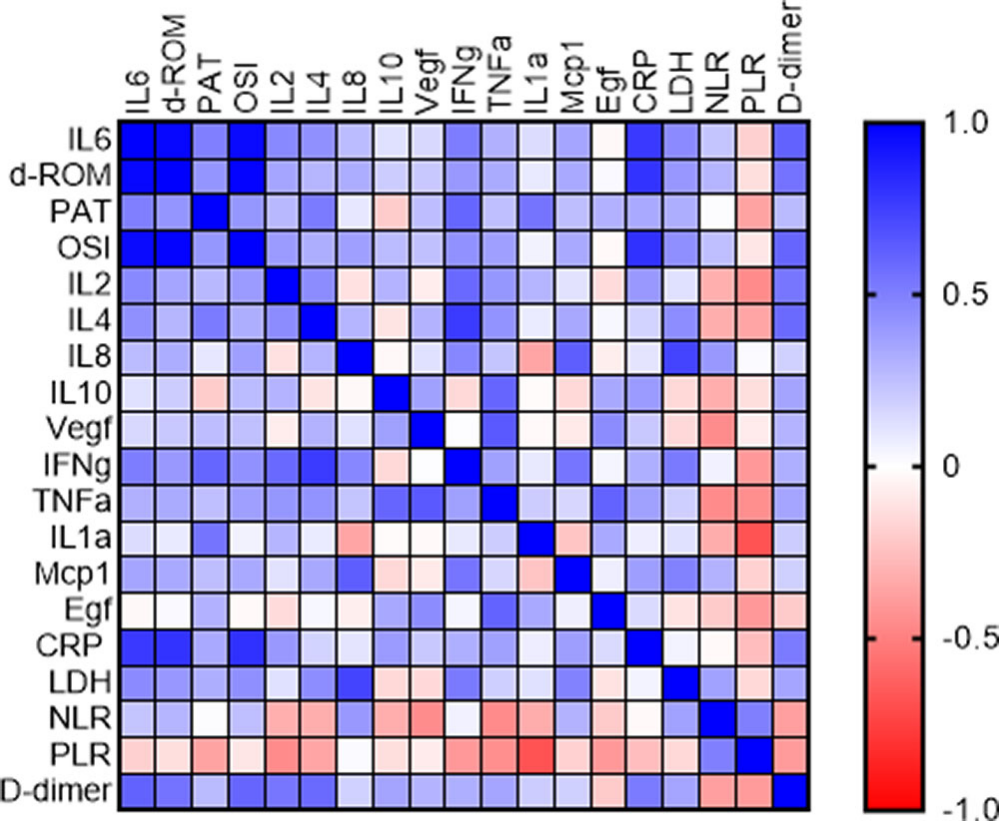
Spearman r presented as a heatmap between the investigated cytokines, oxidative stress parameters, and commonly used biomarkers in COVID-19.

As presented in Table 1, 11 cytokines (including chemokines and growth factors) were analyzed in 14 patients infected with SARS-CoV-2 with severe form of the disease and these values were compared with individuals without SARS-CoV-2 infection. In this comparison, statistically significant increase (p < 0.05, t-test) was observed for IL-6, IL-8, IL-10, VEGF, MCP-1 and EGF in the SARS-CoV-2 patients, while IL-2, IFN-γ, TNF-α and IL-1α were increased but this difference was not significant when compared to the individuals without SARS-CoV-2 infection (p < 0.05, t-test). Important finding of this pilot study is that the parameters of the oxidative stress, d-ROM (448.8 ± 30.37 U.Carr), OS index (107.7 ± 14.38) and PAT (3048 ± 100.1 U.Carr) were significantly higher (p < 0.05, t-test) in severe COVID-19 patients when compared to the not infected individuals ( Table 1). Moreover, we have investigated the correlation among the investigated cytokines, the oxidative stress parameters and CRP, LDH, PLR, D-dimer and NLR. The Spearman r coefficient of correlation between all these parameters is presented as a heat-map on Figure 2. The heat-map confirmed a positive and significant correlation between all cytokines and the parameters of the oxidative stress (d-ROM, PAT and OSI), except a negative correlation between IL-10 and the total antioxidant capacity, PAT. The correlation was not considered to be significant between OS index and the IL-8 (r = 0.3762, p = 0.8552) and between d-ROM and VEGF (r = 0.2156, p = 0.999). IL-6 demonstrated strongest correlation with all of the markers of the oxidative stress, d-ROM (r = 0.9725, p = 0.0001), PAT (r = 0.5000, p = 0.0001) and OS index (r = 0.9593, p = 0.012). Alongside, similar behavior was evidenced between IFN-γ and d-ROM (r = 0.4006, p = 0.0001), PAT (r = 0.6030, p = 0.0001) and OS index (r = 0.4298, p = 0.012). We further investigated the correlation between the cytokines and CRP as one of the most commonly used biomarkers, where the strongest one was observed with IL-6, IL-8, MCP-1 and IFN-γ. Moreover, in terms of correlation, investigated inflammatory cytokines IL-2, IL-4, IL-6, IL-8, IL-10, VEGF, IFN-γ, TNF-α, IL-1α and MCP-1 showed a strong positive correlation between each other, except between IL-6 and EGF ( Figure 2).

## 4. Discussion

Cytokines including chemokines and growth factors together with lipid metabolites are among the main factors of immune cell function and their differentiation, hence upon their dysregulation various diseases can arise. ^7- 9, 12^ Herein, we share our results to give an add-on to the clinical evidences that oxidative stress is increased in patients with severe form of COVID-19 and that the measured oxidative stress parameters had shown a good correlation with the cytokines and the commonly used laboratory biomarkers. This pilot study focused on the possibility to utilize the oxidative stress parameters (d-ROM, PAT and OS index) as a fast and inexpensive prognostic tool for disease progression and potentially predict the outcome of COVID-19 in patients. Several retrospective studies and reviews have been published where abnormal levels of cytokines involved in the adaptive immunity (IL-2, IL-4) or pro-inflammatory cytokines and interleukins (IFNs, IL-1, IL-6, IL-10 IL-17 and TNF-α) were reported. ^7, 13, 18, 19^

Our study revealed that several cytokines and biomarkers were significantly increased in infected SARS-CoV-2 patients with severe form of the disease in comparison to those who were not, which was accompanied with coagulopathy as determined by deterioration of the platelet related parameters (PLR, D-dimer, IL-6) and MCP-1 as thrombosis related indicator. Huang *et al.* (2020) reported that MCP-1 levels were much higher in critical ICU patients and additionally that the platelet count was lower in those patients that do not survive. ^19^ Patients from our study were all with severe form of COVID-19 and all of them had died during hospitalization. Moreover, in our patients several of the cytokines had been increased more than 10-fold above the levels of the non-infected that we considered as a baseline. It is worth noting, the statistically significant increase of the VEGF levels more than 10-fold that can be related to the essential role of VEGF in endothelial cell activation by binding to cell surface VEGF receptors. VEGF up-regulation was observed in several viral infections and it has been investigated as a target for potential therapy development. ^20^ In addition, Huang *et al*., report higher levels of VEGF in hospitalized COVID-19 patients. ^19^ Tanacan *et al.*, had investigated the cytokine profile in pregnant women with COVID-19 where their results indicated significantly higher values for IFN-g andIL-6, and lower values of IL-2, IL-10 and IL-17, especially in those where complications like miscarriage and preterm delivery was evidenced. ^21^

The strong correlation between the investigated cytokines (including chemokines and growth factors), the oxidative stress parameters and some of the commonly used biomarkers (CRP, D-dimers, NLR, PLR) are in line with the proposed cytokine storm as underlying mechanism of the infection. The cytokine storm syndrome occurs when large numbers of leukocytes are activated and release a high concentration of proinflammatory cytokines, with IL-6, IL-10, IFN, MPC-1, IL-1, IL-2 and IL-8 being the foremost. Generally, SARS-CoV-2 infection is associated with oxidative stress, the proinflammatory state, cytokine production, and cell death demonstrated by increase in ROS levels and an alteration of antioxidant defense during the infection. ^11, 22^

Even though limited published data are available, we believe that SARS-CoV-2 in line with other RNA viruses triggers oxidative stress by disturbing the pro-antioxidant–antioxidant balance. ^8, 23, 24^ We have demonstrated the significantly higher level of the d-ROM and OS index values in the infected patients with SARS-CoV-2 when compared with those who were not infected, supporting the hypothesis that viral infection will increase the oxidative stress and complicate the course of the disease. Whilst we consider that the OS index value presents an important parameter that we can have an impact on against COVID-19, by supplementation with antioxidants especially when there is applicable knowledge for several nutraceuticals/vitamins (vitamin C, vitamin D, curcumin, selenium, quercetin and other polyphenols) with proven anti-inflammatory, antioxidant and antiviral capacity. ^25, 26^ Clinical trials of several monoclonal antibodies (Tocilizumab, a monoclonal antibody IL-6 receptor antagonist; Sarilumab – IL-6 antagonist; Anakinra, a recombinant IL-1 Receptor antagonist) against selected cytokines are underway, some of which have already been published. ^27- 29^ Although, data on their effectiveness are emerging additional studies are needed for those treatments to be given routinely in COVID-19.

There are several limitations of the study besides being a single-center experience and a pilot study with only severe and critically ill patients. The herein presented patients were hospitalized at the beginning of the global pandemic when no specific and official guidelines were issued and available to assist the need for hospitalization. They had symptoms developed several days prior being hospitalized, however we believe that these symptoms were not life threatening and the hyper-inflammatory phase was at its beginning stage which is deemed by the obtained levels of the cytokines and the oxidative stress index. Nevertheless, further studies concerning COVID-19 patients with high levels of d-ROMs and OS index are warranted to determine whether supporting antioxidant therapy can reduce the possibility for the fatal outcome of the critically ill COVID-19 patients.

## 5. Conclusion

Our study demonstrates that the levels of IL-6, IL-8, IL-10, VEGF, MCP-1 and EGF were significantly increased in the severe COVID-19 patients. Among the tested correlations between the oxidative stress parameters and the cytokines, the strongest one was between oxidative stress index and IL-6 (r = 0.9593, p = 0.012). The presented results will contribute to support the evidences that the cytokine storm syndrome lies as an immune-pathogenesis during SARS-CoV-2 infection. By using the oxidative stress parameters (d-ROM, PAT, OS index) physicians can provide timely and early interventions in COVID-19 patients. Namely, we consider that the investigated parameters can be used as a tool at the beginning of COVID-19 disease for the general assessment of oxidative stress and hence enabling a better triage of the patients in terms of disease severity.

## Author contributions

MP, DZ, EA contributed to the conception and design of the study. MP and DZ contributed to the oxidative stress parameters analyses, collated the data for the study, and completed all statistical analysis of data. MP wrote the first draft of the manuscript. AE performed the cytokine assay. KS and EA contributed to the clinical evaluation and medical data collection from the COVID-19 patients. All authors read and approved the final version of the manuscript.

## Data availability

### Underlying data

DataDryad: Underlying data for ‘Presentation of cytokine profile in relation to oxidative stress parameters in patients with severe COVID-19: an observational pilot study’. https://doi.org/10.5061/dryad.gf1vhhmqg.

Data are available under the terms of the Creative Commons Zero “No rights reserved” data waiver (CC0 1.0 Public domain dedication).
