# Supplementary material for: Presentation of cytokine profile in relation to oxidative stress parameters in patients with severe COVID-19: a case-control pilot study
Source: F1000Res. 2021 Oct 14;10:719. Originally published 2021 Aug 2. [Version 2] doi: 10.12688/f1000research.55166.2 (PMC8603313; doi:10.12688/f1000research.55166.2)
Supplement: Peer review history

# Peer review: Reviewer response for version 2

**DOI:** 10.5256/f1000research.78311.r96938

**Published:** 2021-11-18

**Authors:** Atakan Tanacan

**Affiliations:** 1 Department of Obstetrics and Gynecology, Turkish Ministry of Health, Ankara City Hospital, Ankara, Turkey

The authors have performed the recommended revisions the manuscript is now appropriate for indexing.

Is the work clearly and accurately presented and does it cite the current literature?

Yes

If applicable, is the statistical analysis and its interpretation appropriate?

Yes

Are all the source data underlying the results available to ensure full reproducibility?

Yes

Is the study design appropriate and is the work technically sound?

Yes

Are the conclusions drawn adequately supported by the results?

Yes

Are sufficient details of methods and analysis provided to allow replication by others?

Yes

Reviewer Expertise:

Perinatology, maternal-fetal medicine, prenatal diagnosis

I confirm that I have read this submission and believe that I have an appropriate level of expertise to confirm that it is of an acceptable scientific standard.

# Peer review: Reviewer response for version 2

**DOI:** 10.5256/f1000research.78311.r96939

**Published:** 2021-10-19

**Authors:** Samuel Asamoah Sakyi

**Affiliations:** 1 Department of Molecular Medicine, School of Medicine and Dentistry, College of Health Sciences, Kwame Nkrumah University of Science and Technology, Kumasi, Ghana

The authors have addressed all my comments and concerns.

Is the work clearly and accurately presented and does it cite the current literature?

Yes

If applicable, is the statistical analysis and its interpretation appropriate?

Yes

Are all the source data underlying the results available to ensure full reproducibility?

Partly

Is the study design appropriate and is the work technically sound?

Partly

Are the conclusions drawn adequately supported by the results?

Yes

Are sufficient details of methods and analysis provided to allow replication by others?

Yes

Reviewer Expertise:

Infectious and non-communicable diseases, biomarkers discovery, (diabetes, hypertension, kidney disorders, cytokines)

I confirm that I have read this submission and believe that I have an appropriate level of expertise to confirm that it is of an acceptable scientific standard.

# Peer review: Reviewer response for version 1

**DOI:** 10.5256/f1000research.58720.r94846

**Published:** 2021-10-07

**Authors:** Samuel Asamoah Sakyi

**Affiliations:** 1 Department of Molecular Medicine, School of Medicine and Dentistry, College of Health Sciences, Kwame Nkrumah University of Science and Technology, Kumasi, Ghana

The current study examined the levels of cytokines and oxidative stress markers among COVID-19 patients and correlated them with disease severity.

 The manuscript is well written except that in the Methods section the authors failed to state the study design. I inferred from the statistical section that the study design was a case-control study. The authors should describe the control very well - how they were recruited to ensure they were devoid of confounding factors, more especially since cytokines are involved. Cytokines are affected by many factors. Additionally, conclusion statements should explain the statistical terms correlation.

Is the work clearly and accurately presented and does it cite the current literature?

Yes

If applicable, is the statistical analysis and its interpretation appropriate?

Yes

Are all the source data underlying the results available to ensure full reproducibility?

Partly

Is the study design appropriate and is the work technically sound?

Partly

Are the conclusions drawn adequately supported by the results?

Yes

Are sufficient details of methods and analysis provided to allow replication by others?

Yes

Reviewer Expertise:

Infectious and non-communicable diseases, biomarkers discovery, (diabetes, hypertension, kidney disorders, cytokines)

I confirm that I have read this submission and believe that I have an appropriate level of expertise to confirm that it is of an acceptable scientific standard, however I have significant reservations, as outlined above.

# Response

**Published:** 2021-10-08

**Authors:** Marija Petrushevska

**Affiliations:** University of Ss Cyril and Methodius, Faculty of Medicine, Macedonia

Thank you for reviewing our manuscript and for your contribution to increasing the quality of our manuscript. We have accepted all your remarks, and we made changes in the material section in order to describe the control (recruitment, risk factors). All changes are marked in red and with track changes. 

 Additionally, in the Section Conclusion - we have elaborated in detail on the statistical correlation.

# Peer review: Reviewer response for version 1

**DOI:** 10.5256/f1000research.58720.r94168

**Published:** 2021-09-21

**Authors:** Atakan Tanacan

**Affiliations:** 1 Department of Obstetrics and Gynecology, Turkish Ministry of Health, Ankara City Hospital, Ankara, Turkey

I have read the manuscript with great interest. The authors have made a comprehensive study on an important topic. I have some minor recommendations for the authors:

1. The inflammatory background of COVID-19 should be discussed.
2. Treatment options and targeted therapies should be discussed in more detail.
3. Severe COVID-19 complications should be discussed in more detail.
4. The clinical implication of findings obtained from the present study should be underlined.
5. The following literature by Tanacan *et al*. 2021 ^1^ may be useful and in my opinion, the mechanisms behind the possible immunologic processes behind COVID-19 may help the authors to improve their manuscript.

Is the work clearly and accurately presented and does it cite the current literature?

Yes

If applicable, is the statistical analysis and its interpretation appropriate?

Yes

Are all the source data underlying the results available to ensure full reproducibility?

Yes

Is the study design appropriate and is the work technically sound?

Yes

Are the conclusions drawn adequately supported by the results?

Yes

Are sufficient details of methods and analysis provided to allow replication by others?

Yes

Reviewer Expertise:

Perinatology, maternal-fetal medicine, prenatal diagnosis

I confirm that I have read this submission and believe that I have an appropriate level of expertise to confirm that it is of an acceptable scientific standard, however I have significant reservations, as outlined above.

## References

[1] : The impact of COVID-19 infection on the cytokine profile of pregnant women: A prospective case-control study. *Cytokine* .140: 10.1016/j.cyto.2021.155431 155431 10.1016/j.cyto.2021.155431 33503581PMC7810028

# Response

**Published:** 2021-09-27

**Authors:** Marija Petrushevska

**Affiliations:** University of Ss Cyril and Methodius, Faculty of Medicine, Macedonia

Thank you for reviewing our manuscript and for your contribution to the increase in the quality of the manuscript. Below I attend to all of your comments. The changes will be included in the 2 ^nd^ version of the manuscript after we receive an additional report from another reviewer. 

**Comment 1, Comment 2, and Comment 5:**

 The authors have discussed the inflammatory background of the disease in the *Section Discussion* on page 6 of 10 (first paragraph). A revision will be inserted where the reference Tanacan *et al*. 2021 will be included, additionally, in this section, we will discuss treatment options and targeted therapies.

**Comment 3:**

 COVID-19 complications are also presented and well referenced in the manuscript. We believe that this has been a subject of numerous publications in the past year. Also, we discussed the most prominent and disturbing symptoms and their onset followed by the clinical biochemical parameters.

**Comment 4:**

 The clinical implication of findings has been pointed out in the S *ection Conclusion*. However, we will elaborate in more detail as stated below:

 "The presented results will contribute to supporting the evidence that the cytokine storm syndrome lies as an immunopathogenesis during SARS-CoV-2 infection. By using the oxidative stress parameters (d-ROM, PAT, OS index) physicians can provide timely and early interventions in COVID-19 patients. Namely, we consider that the investigated parameters can be used as a tool at the beginning of COVID-19 disease for the general assessment of oxidative stress and hence enabling a better triage of the patients in terms of disease severity."
